# Tracking Heat Stress in Broilers: A Thermographic Analysis of Anatomical Sensitivity Across Growth Stages

**DOI:** 10.3390/ani15152233

**Published:** 2025-07-29

**Authors:** Rimena do Amaral Vercellino, Irenilza de Alencar Nääs, Daniella Jorge de Moura

**Affiliations:** School of Agricultural Engineering, State University of Campinas, Av. Cândido Rondon, 501, Campinas 13083-875, SP, Brazil; rimena.vercellino@gmail.com (R.d.A.V.); irenilza@unicamp.br (I.d.A.N.)

**Keywords:** surface temperature, thermal regulation, hyperthermia, precision livestock farming, precision nutrition

## Abstract

This study aimed to identify anatomical regions and developmental stages in broiler chickens that serve as reliable thermographic indicators of acute heat stress. Broilers aged 14, 21, 35, and 39 days were exposed to controlled heat stress, and surface temperatures across 12 anatomical regions were recorded using infrared thermography. Thermal response metrics (maximum, minimum, and mean peak variation) were analyzed with repeated-measures ANOVA and eta squared (η^2^) to quantify the strength of physiological responses. Principal component and cluster analyses grouped body regions based on their thermal sensitivity. The comb and wattle consistently showed the highest temperature increases (ΔT = 2.3–4.1 °C) and strongest effect sizes (η^2^ ≥ 0.70), establishing them as primary thermoregulatory markers. As age increased, more body regions—especially peripheral zones like the drumstick and tail—exhibited strong responses (η^2^ > 0.40), indicating an expansion of thermoregulatory activity. Cluster analysis identified three distinct sensitivity groups, confirming anatomical differences in thermal regulation. Thermographic responses to heat stress in broilers depend on age and region. The comb and wattle are the most reliable biomarkers, while peripheral responses grow more prominent with maturity. These findings support the use of targeted, age-specific infrared thermography for monitoring poultry welfare.

## Simple Summary

This study examined how different parts of the broiler chicken’s body respond to acute heat stress at various stages of growth. Using infrared thermography, surface temperatures were measured in 12 body regions of broilers aged 14, 21, 35, and 39 days. Results showed that the comb and wattle consistently exhibited the highest temperature increases, making them reliable indicators of heat stress. As the birds matured, other areas, mainly peripheral regions, such as the drumstick and tail, also exhibited stronger thermal responses. The findings indicate that heat stress responses in broilers differ by age and body region, supporting the use of infrared thermography for targeted monitoring of poultry welfare.

## 1. Introduction

Heat stress is a major environmental challenge in broiler production, especially under intensive farming conditions where high stocking densities and rapid growth increase the risk of hyperthermia [1,2]. Broilers lack sweat glands and are covered in feathers, making them highly vulnerable to high ambient temperatures that negatively impact growth performance, welfare, immunity, feed intake, and meat quality [3,4]. As global temperatures rise, the need to develop effective strategies to mitigate heat stress becomes increasingly urgent [5]. Modern broiler breeds, which have been intensively genetically selected for productivity, have decreased thermoregulatory ability and show little adaptation to tropical or subtropical climates [6,7].

Infrared thermography (IRT) is a valuable, non-invasive tool for assessing thermoregulatory dynamics in broiler chickens, as it detects changes in surface temperature that correlate with core body temperature and physiological stress markers [5,8,9]. This technology effectively identifies thermally reactive regions, such as the comb, wattle, and legs, which serve as vascular heat exchange zones during stress [8]. It also functions as a practical indicator for evaluating broiler thermal comfort amid heat stress conditions [10]. Surface temperature as a thermal indicator varies as broilers mature. Research indicates that surface temperature (ST) increases with age and in response to higher temperature-humidity indices, suggesting that older broilers may be more susceptible to heat stress or exhibit different thermoregulatory responses [11]. The effectiveness of surface temperature as an indicator may also be influenced by behavioral and physiological changes occurring as broilers develop [12].

Despite its potential, most IRT studies to date have focused on isolated body regions or specific age groups, overlooking the developmental changes that influence thermoregulatory capacity. Anatomical differences in feather coverage and vascularization cause variations in thermal sensitivity throughout the broiler’s growth period [13]. Furthermore, the dynamic interaction between environmental temperature and surface heat flow differs between feathered and unfeathered regions, emphasizing the need for age-specific evaluations [14].

In tropical poultry systems, accurate monitoring of thermal responses is essential for optimizing housing conditions and implementing effective welfare measures. Age and genotype significantly influence surface temperature patterns across various body regions, indicating that targeted thermographic monitoring can enhance the early detection of thermal imbalance [15].

Most studies focus on surface temperature alone or along with a few physiological markers [2]. Further research is required to integrate surface temperature data with behavioral, environmental, and other physiological indicators for a comprehensive evaluation of broiler welfare under heat stress [11,16]. This is the first study to systematically evaluate thermal sensitivity and recovery across multiple anatomical regions and developmental stages in broiler chickens, offering a standardized framework for age-specific thermographic biomarkers of heat stress.

This study aimed to identify age-specific anatomical regions that serve as reliable thermographic biomarkers of acute heat stress in broiler chickens. Using infrared thermography, we measured surface temperature responses across various body regions and developmental stages (14–39 days), with a focus on thermal sensitivity and recovery patterns. By analyzing region-specific heat response trends, the goal was to determine which anatomical sites most accurately indicate thermoregulatory stress and resilience, thereby supporting precise, non-invasive welfare monitoring protocols in broiler production systems.

## 2. Materials and Methods

The experiment was conducted in an environmental chamber divided into three compartments (C1, C2, and C3), each measuring 1.6 × 1.4 × 3.0 m, with 25 birds in each compartment. The birds had an acclimation period before the heat-stress trials. Environmental control and the recording of environmental variables were performed automatically using dedicated software. For each compartment, temperature (°C) and air exchange could be controlled. Simultaneously, automatic control of relative humidity (RH, %) and lighting was maintained across all three compartments.

Air temperature was regulated using two heaters, including a quartz heater (400 W/800 W, AQ01, Ventisol, Araranguá, SC, Brazil) and a fan heater (750 W/1500 W, A1-01, Ventisol, Araranguá, SC, Brazil), along with a split-type air conditioner (9000 BTUs, AS09UB, Samsung, Campinas, SP, Brazil) and a temperature sensor (±0.6 °C, −55 °C to +150 °C, LM35, Texas Instruments, Dallas, TX, USA) installed at a height of 0.40 m from the ground. One axial fan (40 cm, 1/4 hp, 185 W, Tron, Araranguá, SC, Brazil) was installed in each compartment on the side opposite the air inlet, operating at a flow rate calculated to maintain the temperature and humidity levels managed by the software. 

Relative humidity (RH) was controlled by two dehumidifiers (36 W, 4 m^3^ capacity, Desidrat Mini I, Thermomatic, São Paulo, SP, Brazil). These dehumidifiers were placed in each compartment, along with a specific sensor (±3.5%, 0–100%, HIH-4030, Honeywell, Charlotte, NC, USA) located in the central compartment (C2), also installed at a height of 0.40 m from the ground floor.

Water and commercial feed, based on corn and soybean meal, were provided ad libitum throughout the entire rearing period. Feed management and drinker cleaning were performed twice daily—once in the morning and once in the late afternoon.

The lighting program consisted of 24 h of light during the first week, 1 h of darkness in the second week, and 4 h of darkness from the third week onward, following current standards used in the poultry industry. The light intensity applied was 25 lx until the third week, 15 lx until the fourth week, and 5 lx from the fifth week onward. Both the lighting schedule and light intensity were based on current poultry industry standards.

Infrared thermal images were collected at three distinct time points: before the onset of stress, during the stress period, and after the stress period had ended. In both experiments, five birds (Cobb^®^ strain) from each of three compartments where the treatments were divided and randomly selected at 14 and 21 days of age (Experiment I) and 35 and 39 days of age (Experiment II). In Experiment I (14 and 21 days of age), thermal images were captured every 120 min during the stress period at 120, 240, 480, and 600 min. In Experiment II (35 and 39 days of age), images were taken at 90-min intervals, specifically at 90, 180, 270, 360, and 450 min. This staggered timing allowed for more frequent monitoring in older birds, while also maintaining overlap around mid-stress exposure points (e.g., 240–270 and 450–480 min), enabling comparative analyses across age groups.

**Figure 1:**
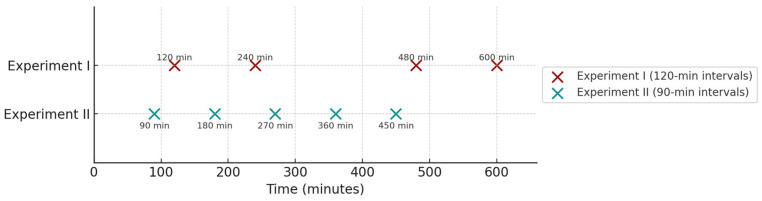
Timeline of thermographic image collection during acute heat stress trials in broiler chickens. Experiment I (14 and 21 days of age) involved image acquisition every 120 min, while Experiment II (35 and 39 days of age) used 90-minute intervals. Overlapping time points around 240–270 and 450–480 min allowed for comparative analysis across developmental stages. This sampling structure facilitated age-specific evaluation of thermal responses.

After the stress period ended, images were captured during the final hour of stress and then every 30 min for a total of two hours. Thus, for Experiment I, images were taken at 720 min (or the 12th hour) of stress and 30, 60, 90, and 120 min post-stress. For Experiment II, images were taken at 450 min (or the 9.5th hour) of stress and also at 30, 60, 90, and 120 min after the stress ended. A total of 15 thermographic images were taken for each body region and age. Figure 1 illustrates the timeline of the collections for both experiments.

Experiment I was conducted when the birds reached 7, 14, and 21 days of age. At these ages, the birds were exposed to 12 h of stress, starting from the moment the target temperatures in each compartment were reached. Compartment T1 received the control treatment, T2 the cold stress treatment, and T3 the heat stress treatment (Table 1).

The treatments included the following: (T1) a thermoneutral environment, considered the control; (T2) a cold stress environment, with temperatures 4 °C below the thermoneutral temperature; and (T3) a heat stress environment, with temperatures 4 °C above the thermoneutral temperature (Table 2). 

In Experiment 2, the treatments consisted of the following: (T1) a neutral (or control) environment with a thermoneutral temperature (21.0 °C ± 1.0 °C); (T2) an environment with moderate heat stress (28.0 °C ± 1.0 °C); and (T3) a severe heat stress environment (30.0 °C ± 1.0 °C). Compartment T1 received the control treatment (thermoneutral environment), T2 received the moderate heat stress treatment, and T3 received the severe heat stress treatment (Table 2).

**Figure 2:**
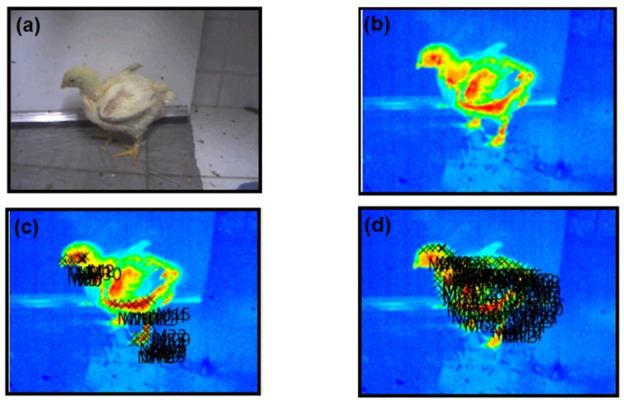
Arrangement for obtaining the surface temperature of each bird in Experiment I: (**a**) real image of the bird; (**b**) thermographic image; (**c**) areas of the body surface without feathers and their respective points; and (**d**) areas of the body surface with feathers and their respective points (only one age is being represented). Red color is related to high temperature and blue colors are related to low temperatures.

**Figure 3:**
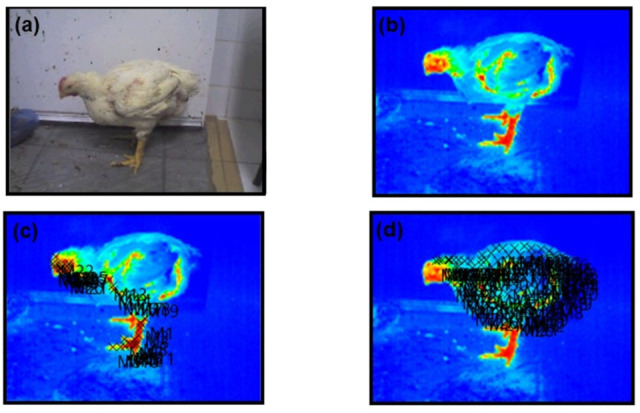
Arrangement for obtaining each bird’s surface temperature in Experiment II: (**a**) real image of the bird; (**b**) thermographic image; (**c**) body surface regions without feathers and their respective points; and (**d**) body surface regions with feathers and their respective points (only one age is being represented). Red color is related to high temperature and blue colors are related to low temperatures.

For each bird, a complete lateral profile photo was obtained (Figure 2a,b) using an infrared thermographic camera (Testo 800, Testo SE & Co. KGaA, Lenzkirch, Germany) equipped with a 160 × 120-pixel uncooled microbolometer sensor, thermal sensitivity <0.1 °C at 30 °C, spectral range of 8–14 µm, and accuracy of ±2.0 °C of the reading. The camera was calibrated before each session and operated at a fixed distance of approximately 1.0 m under consistent ambient lighting conditions to ensure image quality and measurement reliability. The images were analyzed using the camera’s software (Testo IRSoft Software version 5.2, Testo SE & Co. KGaA, Lenzkirch, Germany), with the cold/hot palette option selected (Figure 2b and Figure 3b) [18].

To assess ST, each bird’s body was divided into 14 regions, further classified into feathered and non-feathered areas, with emissivity indices (ε) of 0.95 and 0.94, respectively. The ST of each region was determined by selecting a predefined number of random points. From each anatomical region, 10 pixel points were randomly selected using Python v. 3.10.12 ‘random. Sample’ function with a fixed seed of 42 to ensure reproducibility. For non-feathered regions (Figure 2c and Figure 3c), the analyzed measurements included the comb, eye, ear, and wattle (each at three points), the area under the wing (at seven points), and the foot (at twelve randomly selected points). For feathered regions (Figure 2d and Figure 3d), the following were included: head (4 random points), neck (12 points), back (15 points), wing (24 points), breast and thigh (8 points each), drumstick (9 points), and tail base (6 points).

Thermal sensitivity was defined as the degree of surface temperature change in a specific body region in response to acute heat stress. It was quantified as the difference between the average temperature recorded during the heat stress period and the baseline temperature before exposure, as shown in Table 1 and Table 2 (Equation (1)).

ΔT = T_During heat stress − T_Before heat stress(1)

Regions exhibiting larger positive ΔT values were considered thermally sensitive, indicating a greater physiological response to external heat. 

Thermal sensitivity was quantified by calculating the change in surface temperature for each anatomical region across three phases: before, during, and after heat stress exposure. For each body part and age group, the mean temperature was extracted from thermographic images and used to compute three peak variation metrics. Two additional thermal change metrics were calculated: (i) recovery change, the difference between post-stress and stress-phase temperatures (After–During), reflecting the region’s cooling response; and (ii) net thermal change, the difference between post-stress and baseline temperatures (After–Before), representing the overall thermal shift across the exposure and recovery period. A repeated-measures analysis of variance (ANOVA) was conducted to assess statistically significant differences across phases, and effect sizes were calculated using eta squared (η^2^) to evaluate the magnitude of the physiological response. Effect sizes were interpreted according to established thresholds [19,20], categorizing each region by its thermal response intensity (none, slight, moderate, or strong) (Table 3 and Table 4).

Thermal responses for each body region were analyzed using a one-way repeated-measures ANOVA, with thermal phase (Before, During, After) as the within-subject factor and bird ID as the repeated measure. The model assessed differences in surface temperature across phases while accounting for intra-individual variability. When the assumption of sphericity was violated, Greenhouse–Geisser corrections were applied. Statistical significance was set at *p* < 0.05, and effect sizes were reported using η^2^, classified as not meaningful (<0.01), slight effect (0.01–0.06), moderate (0.06–0.14), and strong (>0.14). To support the interpretation of regional thermal dynamics, key thermal response variables were defined and summarized in Table 4. Thermal recovery was calculated as the difference between post-stress and peak stress temperatures (After–During), and net thermal change was calculated as the difference between post- and pre-stress temperatures (After–Before). These metrics were used to evaluate regional cooling efficiency and overall thermal load.

Thermal differences across phases were summarized using recovery change and net thermal change metrics, previously defined to capture post-stress cooling and overall thermal shift, respectively.

### Data Analysis

To identify thermoregulatory patterns across different anatomical regions, a multivariate cluster analysis was conducted using principal component analysis (PCA) followed by K-means clustering. In this analysis, each body region was treated as an observation, described by its mean thermal response (During–Before) at each of the four ages (14, 21, 35, and 39 days), resulting in four features per region. PCA was used to reduce the dimensionality for visualization, followed by K-means clustering to group body regions with similar age-dependent thermal sensitivity patterns. A K-means clustering algorithm (k = 3) was applied to the PCA-transformed data to identify groups of body regions with similar thermal response patterns across different age groups. The number of clusters was chosen based on interpretability and visual inspection of the PCA output.

Prior to PCA, surface temperature differences (ΔT) were calculated for each body region and age group between the heat stress and baseline phases. All ΔT values were standardized using z-score normalization to eliminate unit bias and ensure comparability across regions. PCA was then applied to reduce dimensionality while retaining the majority of data variance. The first two principal components, explaining 58.3% and 24.7% of the variance, respectively, were used as inputs for clustering. The optimal number of clusters (k = 3) was determined using the elbow method and silhouette analysis, which balances interpretability with model fit, thereby ensuring a suitable balance between the two.

A one-way repeated-measures ANOVA was performed for each body region to assess differences in surface temperature across the three thermal exposure phases (Before, During, and After heat stress). The repeated-measures structure accounted for within-subject variability, as each bird contributed temperature data for all three phases. The model included ‘Phase’ as the within-subject factor, and bird ID was treated as the repeated subject identifier. Assumptions of sphericity were tested using Mauchly’s test, and when violated, Greenhouse–Geisser corrections were applied. Effect sizes were reported using eta squared (η^2^). For all analyses, we defined statistical significance as *p* < 0.05, with *p* < 0.01 considered highly significant and 0.05 ≤ *p* < 0.10 indicating a trend. We used Python v. 3.10.12 software with the following libraries to process the data: Pandas, NumPy, Matplotlib, Seaborn, scikit-learn, SciPy, and Random.

## 3. Results

The thermographic analysis revealed clear, age-related patterns of surface temperature changes across different anatomical regions, indicating varied thermal sensitivity and recovery responses to acute heat stress.

### 3.1. Broiler Thermal Response Descriptive Analysis

This section provides a detailed analysis of surface temperature responses in broiler chickens exposed to acute heat stress at four key developmental stages: 14, 21, 35, and 39 days of age. For each anatomical region, thermal sensitivity was evaluated using peak temperature changes and effect size (η^2^) estimates derived from repeated-measures analysis of variance (ANOVA). These data offer insights into how physiological heat regulation develops with age and varies across body regions. Table 3, Table 4, Table 5 and Table 6 summarize the mean temperatures, statistical significance, and the magnitude of thermal responses, offering a comprehensive overview of anatomical and developmental differences in broiler thermoregulation. Detailed information on mean values and standard errors is available in the Supplementary Materials (Tables S1–S4).

The thermal response data for 14-day-old broilers (Table 5) show distinct variations in surface temperature across body regions during the heat stress protocol. Among all evaluated regions, the comb, wattle, eye, leg, and tail exhibited the strongest thermal responses, with temperature increases ranging from approximately 2.4 °C to 3.7 °C and η^2^ values above 0.14, indicating they are strongly affected by heat stress. These regions are likely involved in active thermoregulation and serve as reliable markers for detecting heat stress in early development. Moderate physiological responses were observed in regions such as the neck, flank, and drumstick, with η^2^ values ranging from 0.06 to 0.14. These areas showed less pronounced temperature changes, suggesting a supportive role in heat dissipation. Regions like the head, ear, back, breast, thigh, and wing exhibited only minimal or no responses, with low effect sizes (η^2^ < 0.06) and small peak temperature shifts. The wing showed no statistically significant change (*p* = 0.814, η^2^ = 0.001), emphasizing its limited role in acute thermal response at this age.

At 21 days of age (Table 6), broilers still showed apparent regional differences in their thermal response to heat stress. The comb, wattle, ear, leg, and thigh continued to display high sensitivity, with surface temperature increases exceeding 2 °C and η^2^ values consistently above 0.50, indicating a strong physiological effect. These areas are thermally reactive and remain reliable indicators of heat stress during this growth stage. The breast, back, and flank exhibited negative ΔT values, indicating surface cooling during stress. Despite this decrease, several of these regions still showed statistically significant changes (*p* < 0.05), suggesting an active physiological response such as vasoconstriction or evaporative heat loss, rather than a lack of thermoregulatory engagement.

As shown in Table 6, the comb, wattle, eye, back, and leg experienced the highest temperature increases, with peak changes ranging from 3.00 to 5.00 °C and η^2^ values above 0.70, indicating a significant physiological effect. These regions were highly reactive, reflecting vigorous vascular thermoregulation at this stage of development.

Despite this, effect sizes for these areas remained significant, suggesting active physiological responses. The eye, drumstick, and neck showed moderate responses. At the same time, the wing again displayed minimal variation, with the lowest η^2^ (0.044) and no significant temperature change, emphasizing its role as a thermally stable or dissipation region.

Comparing the thermal responses between 14- and 21-day-old broilers reveals a consistent pattern in the most thermally responsive regions, with the comb, wattle, eye, and leg consistently showing the highest temperature increases and strongest statistical effects (η^2^ > 0.50) at both ages. These areas are likely the primary thermal windows involved in regulating acute heat stress during early development. However, at 21 days, the magnitude and statistical strength of these responses generally increased. For example, the leg and wattle exhibited peak changes exceeding 3 °C and η^2^ values above 0.6, indicating increased thermal sensitivity or vascular capacity as the birds aged. Conversely, some regions that showed only moderate responses at 14 days, such as the neck, back, and drumstick, demonstrated stronger physiological engagement by 21 days, possibly reflecting maturation of peripheral thermoregulation mechanisms. Meanwhile, the wing remained consistently unresponsive across both ages, reinforcing its classification as a non-sensitive or thermally neutral zone.

Table 7 and Table 8 display the thermal responses of broilers at 35 and 39 days of age. At 35 days (Table 7), broilers exhibit a consistently strong thermal response across nearly all anatomical regions during heat stress.

The neck, head, flank, and thigh also showed significant increases, with η^2^ values ranging from 0.52 to 0.71, further supporting their role in heat dissipation. Interestingly, even the tail and drumstick, which showed moderate sensitivity at younger ages, now displayed strong thermal responses (η^2^ > 0.40), indicating a broader distribution of thermoregulatory surfaces as broilers age. The ear showed minimal change between phases (Mean Peak = 0.61 °C). However, it remained statistically significant (*p* = 0.044). Meanwhile, the wing was the only region classified as having a moderate physiological response (η^2^ = 0.07, *p* = 0.45), continuing its pattern as a thermally stable or dissipation zone. This consistent pattern across multiple regions reflects a mature and highly active thermoregulatory system in 35-day-old birds. At 39 days of age (Table 6), broilers exhibited broad and strong thermal responses to acute heat stress across nearly all body regions. As shown in Table 6, areas such as the comb, wattle, leg, tail, and flank displayed mean peak temperature increases exceeding 2 °C, with η^2^ values ranging from 0.48 to 0.84, indicating a significant physiological impact. Notably, the leg and wattle stood out as the most thermally responsive areas, with ΔT values of 2.17 °C and 2.34 °C, respectively, and η^2^ approaching 0.85, highlighting their efficiency in vascular heat exchange.

Several other regions, including the back, eye, head, ear, and drumstick, also showed statistically significant and thermally meaningful changes, further supporting their role in active thermoregulation. The breast and wing were the only areas not classified as having a substantial impact. While the breast exhibited a moderate response (η^2^ = 0.10), the wing had the lowest response overall (ΔT = 0.36 °C; η^2^ = 0.03), indicating that it continued to serve as a thermally stable or passive heat dissipation region.

Comparing 35- and 39-day-old broilers shows a consistent pattern in thermal sensitivity, with most areas maintaining or even increasing their response to heat stress as the birds grow. Both age groups exhibit strong effects (η^2^ > 0.50) in key thermoregulatory regions, such as the comb, wattle, leg, and back, highlighting their ongoing role in heat dissipation. At 39 days, however, the strength and spread of the thermal response become more extensive, with additional areas such as the tail, ear, and flank exceeding strong effect thresholds. This outcome suggests a fully developed thermoregulatory system, where peripheral regions become more actively involved. Meanwhile, the wing remains the least responsive region in both groups, reinforcing its status as a poor indicator of thermal conditions [15].

### 3.2. Thermal Sensitivity During Heat Stress

**Figure 4:**
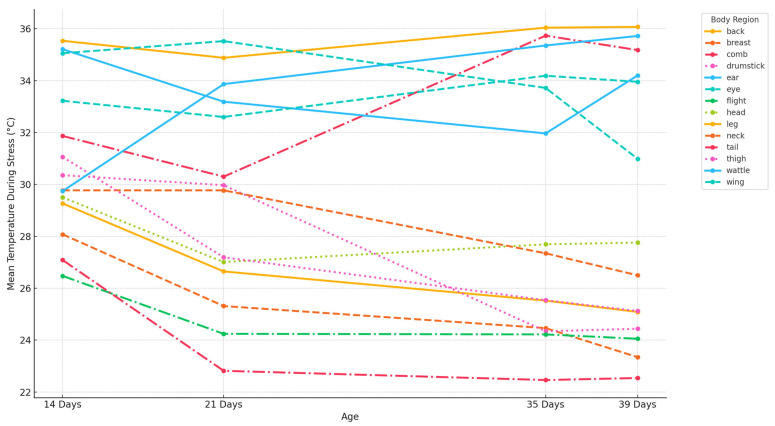
Thermal sensitivity during heat stress in the broiler body regions at different ages.

Figure 4 shows the average surface temperature of various anatomical regions in broiler chickens during the heat stress phase across four growth stages (14, 21, 35, and 39 days of age). The goal was to evaluate the thermal sensitivity of each region, defined as the absolute increase in temperature during acute heat exposure.

The comb and wattle consistently exhibited the highest temperatures, particularly in older birds, highlighting their roles as primary sites for heat dissipation and indicators of thermoregulatory activity. The eye and neck showed moderate increases, indicating intermediate sensitivity. Meanwhile, the feet, wings, and flank maintained lower temperatures across all ages, probably acting as peripheral heat sinks or regions with less vascular thermoreactivity.

The mean peak increase reflects the average surface temperature rise during acute heat stress relative to baseline (Table 4). The comb and wattle showed the highest mean peak increase (ΔT > 2.5°C), indicating these highly vascularized regions are the most thermally sensitive during heat exposure. The minimum and maximum deviation capture individual variability in stress response (min = greatest cooling, max = highest peak). The flank and chest regions showed the broadest range in thermal deviation (ΔT range: −0.8 °C to +2.1 °C), reflecting inter-individual variation in stress buffering capacity.

### 3.3. Heat Map of Thermal Stress

**Figure 5:**
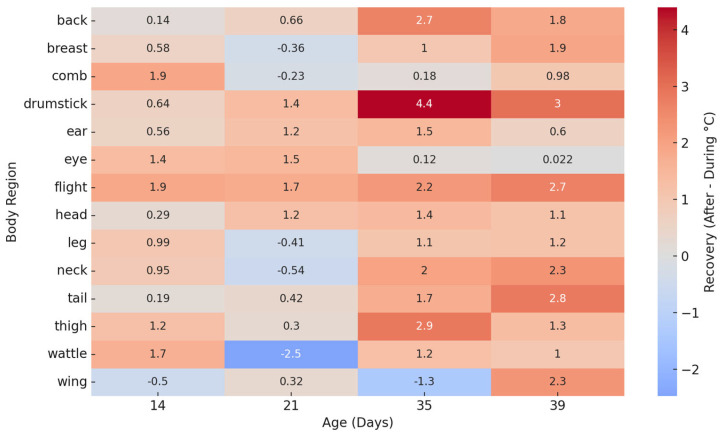
Heatmap showing the thermal recovery capacity (°C) for each body region of broilers at different ages. Recovery was calculated as the difference between surface temperatures after and during heat stress. Positive values indicate a post-stress temperature rebound, while negative values suggest potential cooling or thermal exhaustion.

The heatmap analysis (Figure 5) illustrates the mean temperature increase from baseline (Before) to peak stress (During) across body regions and developmental stages. Consistently across all ages, the comb, wattle, and leg exhibited the highest increases, reinforcing their role as primary thermoregulatory zones. In contrast, regions such as the tail, drumstick, and flank showed minimal or even negative changes, suggesting a buffering or heat-dissipating role. These findings highlight the age-independent thermal sensitivity of specific anatomical regions and support their potential use as markers for the non-invasive detection of heat stress.

### 3.4. Thermal Recovery Capacity

**Figure 6:**
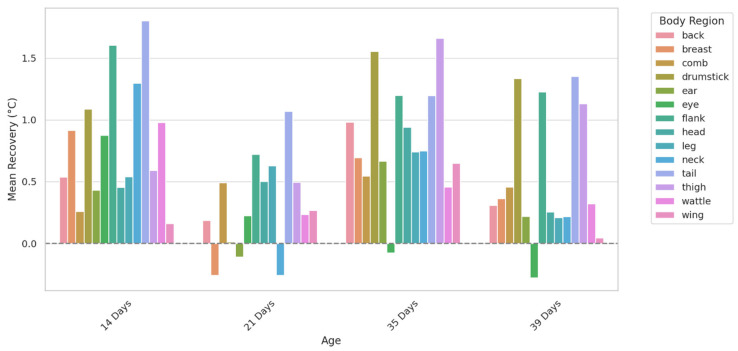
Thermal recovery capacity (after and during heat stress) of broiler chickens across different body regions and ages. The feet region was recorded but not included in this figure due to limited reliability in thermal capture, often influenced by ground contact.

Figure 6 illustrates the thermal recovery response of each body region. The variations suggest they play a central role in heat dissipation after acute stress exposure, with the highest recovery observed in the comb and wattle. Regions such as the back, chest, and flank showed lower thermal recovery values compared to vascularized areas like the comb and wattle, indicating a less pronounced surface temperature increase after heat stress.

Although surface temperatures remained elevated post-stress in comb, wattle, and eye regions, the magnitude of recovery decreased slightly with advancing age, suggesting a potential reduction in peripheral vasodilation capacity or thermal resilience in older broilers. On the 21st day, the overall data decreased compared to days 14, 35, and 39, probably because the birds were in the process of changing their feathers.

### 3.5. Cluster Analysis

**Figure 7:**
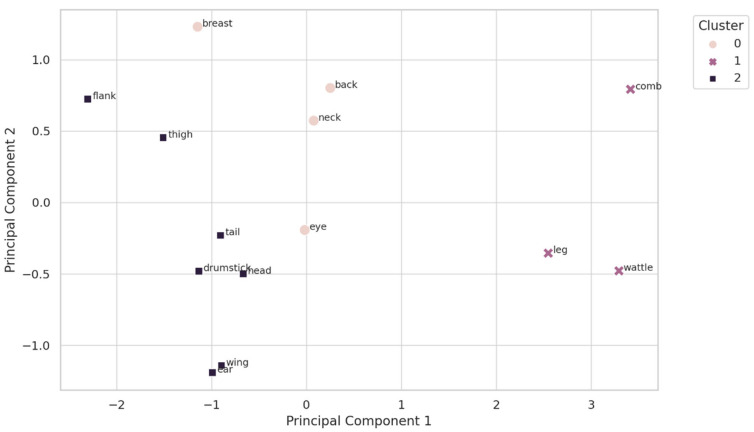
Cluster analysis of broiler body regions based on thermal sensitivity to heat stress. Principal component analysis (PCA) was applied to standardized ΔT values, with PC1 and PC2 accounting for 58.3% and 24.7% of total variance, respectively. K-means clustering (k = 3) grouped body regions by thermal response. Cluster 1 (×, purple) represents high-sensitivity zones (comb, wattle, leg); Cluster 0 (●, pink) includes moderate responders (eye, neck, back, breast); Cluster 2 (■, dark grey) denotes low-sensitivity regions (wing, ear, tail, drumstick, flank). Colors in the plot match the legend for consistent interpretation.

The cluster analysis (Figure 7), visualized through a PCA-based scatterplot, identified three distinct groups of body regions based on their average thermal response to heat stress across developmental stages. Cluster 1 comprised the comb and wattle, which consistently exhibited the highest thermal sensitivity (i.e., the most significant temperature increases during stress), marking them as primary thermoregulatory surfaces and optimal candidates for thermographic monitoring.

Cluster 1: High sensitivity (e.g., comb, wattle)

Cluster 0: Moderate responders (e.g., eye, neck, back)

Cluster 2: Low sensitivity (e.g., wing, feet, flank)

Cluster 0 included regions such as the eye, neck, back, and breast, which showed moderate and consistent temperature elevations, suggesting supporting roles in heat regulation. Cluster 2, encompassing the feet, wings, and flank, was characterized by low or negative thermal shifts, indicating its function as a passive heat dissipation zone or a thermally stable region. This approach enabled the robust classification of body regions into high-, moderate-, and low-sensitivity groups based on their thermal response profiles. This method enabled the identification of high-, moderate-, and low-sensitivity regions, clarifying the roles of different anatomical areas in thermoregulation during acute heat exposure.

## 4. Discussion

This study provides a detailed thermographic profile of broiler chickens exposed to acute heat stress across four developmental stages, highlighting clear age-dependent and anatomical differences in thermal sensitivity and recovery. The integration of surface temperature dynamics with physiological effect size interpretation (η^2^) and multivariate clustering contributes a novel framework for identifying reliable thermoregulatory biomarkers. The findings reinforce the value of peripheral, highly vascularized regions as thermal indicators. Although temperature data from the feet region were collected, this area was excluded from the final thermal recovery analysis due to inconsistent exposure and high variability in temperature readings, likely influenced by direct contact with the ground and shading during imaging. Future studies using standardized positioning or foot elevation may help clarify the thermoregulatory role of this region.

The comb and wattle consistently stood out as the most thermally responsive areas at all ages, showing the most significant surface temperature increases and the strongest effect sizes (η^2^ > 0.70 in older birds). These structures are highly vascularized and minimally insulated by feathers, which supports their role in heat dissipation. Druyan et al. [8] demonstrated that the comb and wattle serve as primary thermal windows due to their rich blood supply. At the same time, Tickle and Codd [21] emphasized that reduced feather insulation enhances radiative and convective cooling efficiency in these regions. Their consistent performance as thermographic markers makes them useful for precise welfare monitoring systems. In contrast, regions such as the wing and flank consistently showed low or negative thermal responses, which often did not reach statistical significance. The lower thermal recovery observed in the back, chest, and flank may reflect reduced peripheral blood flow in these regions or structural limitations in heat dissipation, supporting their role as less responsive thermal windows under acute heat stress.

Thermal recovery, as estimated by the difference between post-stress and peak temperatures, was most prominent in the eye, comb, and wattle, aligning with their role in active thermoregulation. This outcome suggests these areas act more as passive dissipators or thermally stable zones rather than active regulators [15]. Such anatomical stability could help create baseline thermographic maps for diagnosing heat stress.

A key insight from this study is how thermal sensitivity increases with age. Older broilers (35–39 days) exhibit broader and more intense thermoregulatory activity across different body regions, with more areas surpassing the strong-effect threshold (η^2^ ≥ 0.14). This result reflects the physiological development of the cardiovascular and respiratory systems, which enhances their ability to redistribute heat in response to stress [14,15]. The increased involvement of peripheral zones, such as the tail and drumstick, in later stages suggests a progressively expanded thermoregulatory network as broilers approach market weight, likely due to increased peripheral vascularization and enhanced blood flow capacity in response to thermal loads. This physiological adaptation enables greater surface area for heat dissipation and aligns with findings that thermoregulation improves with cardiovascular and integumentary maturation in older broilers [14]. Some regions, such as the neck and back, shift from moderate to strong responders with age, indicating developmental changes in vascular control and surface heat exchange. These dynamics highlight the importance of age-specific thermal reference values when using infrared thermography (IRT) in commercial settings [15]. Although the comb, wattle, and eye regions consistently exhibited positive thermal recovery values across all ages, a slight reduction in recovery magnitude was observed in older broilers, which may indicate age-related declines in peripheral vasodilation efficiency or thermal regulatory capacity.

Thermal recovery data further confirms the diagnostic importance of head-related regions. The comb and wattle not only responded strongly to heat stress but also showed effective post-stress rewarming, indicating both thermosensitivity and resilience [2]. In contrast, ongoing cooling in areas such as the wing and feet suggests that their role may be limited to passive heat dissipation, without significant involvement in post-stress homeostasis [10]. The recovery patterns indicate that thermographic assessments taken immediately after heat exposure can still provide valuable clues about heat load and resilience, especially in vascularized regions.

The cluster analysis reinforced the anatomical separation of thermoregulatory roles. Cluster 1 (comb, wattle, leg) represented high-sensitivity zones ideal for IRT-based monitoring. Cluster 0 (eye, neck, back) included moderate responders, while Cluster 2 (wing, flank, ear) consisted of thermally neutral or dissipative zones. This stratification supports a level-up monitoring approach, prioritizing Cluster 1 for diagnostic accuracy and using Clusters 0 and 2 for contextual calibration [11].

Collectively, the findings emphasize the importance of dynamic, region-specific, and age-adjusted thermographic assessments in broiler management. Standardized heat stress indices must account for anatomical and developmental differences to prevent underestimating or overestimating the thermal load [22,23]. These results provide a foundation for developing automated thermal alert systems in commercial poultry houses, where infrared thermography can be integrated with environmental control technologies, such as sensor-driven ventilation systems [11,16]. For example, real-time IRT data from the comb and wattle could be used to trigger adaptive ventilation rates or localized cooling units, enabling responsive thermal regulation based on the birds’ physiological state rather than ambient conditions alone. This approach enhances welfare monitoring and reduces reliance on static temperature thresholds.

This study, while providing valuable insights into anatomical and age-related thermoregulatory patterns in broilers, has several limitations. First, all experiments were conducted in controlled environments, which, although ideal for standardization, may not fully reflect the complexity of commercial poultry houses where multiple stressors occur. Additionally, the study focused solely on surface temperature data, excluding concurrent physiological (e.g., heart rate, hormone levels) and behavioral indicators that could enhance the interpretation of heat stress responses. The timing of thermal measurements was also limited, as data were collected at specific time points rather than continuously, which could potentially miss transient thermal fluctuations. Moreover, only one commercial broiler genotype was examined, limiting the applicability of the findings to other genetic strains with different thermotolerance profiles. Technical limitations of infrared thermography, such as sensitivity to environmental lighting, feather coverage, and calibration accuracy, may have also introduced variability in surface temperature readings. Finally, the study addressed acute heat stress exposure without considering the effects of chronic or long-term thermal challenges, which are more common in field conditions and may involve different physiological mechanisms.

The findings of this study, based exclusively on Cobb^®^ broilers, may not be fully applicable to other genetic strains due to genetic differences in thermoregulation physiology. Modern broiler lines, such as Cobb^®^, have been heavily selected for rapid growth and muscle development, resulting in increased metabolic heat production and reduced feather coverage. This consequence makes them more sensitive to heat stress and changes in surface temperature [21]. In contrast, slower-growing or heritage strains often exhibit better-developed feathers, lower metabolic rates, and distinct blood flow patterns in their extremities, all of which can impact thermal imaging results and the accuracy of thermographic markers, such as the comb and wattle [8,15]. Additionally, feather growth and vascular responses vary with age and genotype, which can alter the timing and location of thermal responses during stress [13,22]. Therefore, caution should be exercised when applying these thermographic profiles to other genotypes, and further validation studies are necessary to establish strain-specific thermal reference values.

## 5. Conclusions

This study validates that thermographic responses to acute heat stress in broiler chickens are both age-dependent and anatomically specific. Throughout all developmental stages, the comb and wattle consistently exhibited the highest surface temperature elevations and strongest effect sizes, establishing them as robust thermographic biomarkers for heat stress detection. As broilers matured, their thermoregulatory response became more widespread and intensified. Specifically, the number of anatomical regions exhibiting a strong physiological effect (η^2^ ≥ 0.14) increased from 4 at 14 days of age to 12 at 39 days, indicating that nearly the entire body became actively involved in heat dissipation by the end of the growth period. Peripheral regions, such as the tail and drumstick, exhibited marked increases in responsiveness, indicating the maturation of vascular and thermoregulatory capacities.

In contrast, regions like the wing and flank remained thermally stable, underscoring their limited utility for stress monitoring. These findings support the development of precision thermal monitoring strategies that are anatomically targeted and age specific. By identifying consistently responsive regions, such as the comb and wattle, this study provides a scientific basis for integrating infrared thermography into automated broiler welfare monitoring systems, enabling the real-time detection of heat stress and the timely implementation of management interventions in commercial poultry operations.

## Figures and Tables

**Table 1:** Temperature values and stress periods applied at 7, 14, and 21 days of age.

Age (Days)	Temperature (°C)		
	T1 *	T2	T3
	Thermoneutral (Comfort)	Cold Stress	Hot Stress
7	30.0	25.0	33.0
14	27.0	23.0	31.0
21	24.0	19.0	28.0
Stress period (hours)	0	12	12

* Ideal values for each age, obtained and adapted from the strain manual [17].

**Table 2:** Temperature values and stress periods applied at 35 to 39 days of age.

Age (Days)	Temperature (°C)		
	T1 *	T2	T3
	Thermoneutral (Comfort)	Moderate Heat Stress	Severe Heat Stress
35	21.0	28.0	30.0
39	21.0	28.0	30.0
Stress period (hours)	0	8	8

* Ideal values for each age, obtained and adapted from the strain manual [17].

**Table 3:** Interpretation of effect size (η^2^) used to assess thermal response intensity.

η^2^ Range	Effect Size Interpretation	Source
η^2^ < 0.01	No meaningful effect	[20]
0.01 ≤ η^2^ < 0.06	Slight effect of heat stress	[19,20]
0.06 ≤ η^2^ < 0.14	Moderate physiological response	[19]
η^2^ ≥ 0.14	Strong impact of heat stress	[19]

**Table 4:** Definition of the temperature peak and the mean peak outcome.

Temperature	Interpretation
Mean peak outcome	The average of the individual maximum temperature values recorded during the observation period
Mean peak increase	Recovery change (After–During). Mean difference in temperature between stress and baseline across birds.
Maximum individual deviation	Net thermal change (After–Before). The highest recorded change in any individual bird from baseline.

**Table 5:** Thermal response of 14-day-old broiler chickens during acute heat stress *.

Body Region	Mean Temp. Before HS (°C)	SE _Before HS	Mean Temp. During HS (°C)	SE _During HS	Mean Temp. After HS (°C)	SE _After HS	Mean Peak Outcome (°C)	*p*-Value	η^2^	Effect Classification of the Heat Stress
Back	29.71	0.09	30.09	0.11	30.63	0.11	0.92	>0.001	0.06	Slight effect
Breast	30.37	0.15	29.30	0.23	30.23	0.22	−0.15	>0.001	0.04	Slight effect
Comb	30.08	0.26	32.81	0.47	33.07	0.39	2.99	>0.001	0.22	Strong impact
Drumstick	30.84	0.17	31.14	0.21	32.23	0.16	1.39	>0.001	0.08	Moderate physiological response
Ear	35.32	0.13	35.40	0.18	35.83	0.13	0.52	0.037	0.05	Slight effect of heat stress
Eye	34.24	0.12	34.0	0.15	34.87	0.11	0.59	>0.001	0.16	Strong impact
Flank	28.56	0.13	26.765	0.12	28.37	0.11	−0.19	>0.001	0.11	Moderate physiological response
Head	30.33	0.18	30.35	0.22	30.81	0.15	0.48	0.123	0.02	Slight effect
Leg	33.89	0.20	35.76	0.16	36.30	0.13	2.41	>0.001	0.18	Strong impact
Neck	30.85	0.14	30.51	0.15	31.80	0.15	0.95	>0.001	0.08	Moderate physiological response
Tail	26.884	0.26	27.80	0.29	29.59	0.32	2.71	>0.001	0.15	Strong impact
Thigh	31.40	0.17	32.10	0.26	32.69	0.19	1.30	>0.001	0.05	Slight effect
Wattle	28.11	0.34	30.86	0.50	31.84	0.29	3.73	>0.001	0.27	Strong impact
Wing	35.24	0.18	35.18	0.18	35.34	0.18	0.10	0.814	0.00	Not meaningful

* Statistical significance (*p*-value) and effect size (η^2^) are reported based on repeated-measures ANOVA. SE = Standard error. Effect sizes are interpreted using thresholds to classify the physiological impact of heat stress: no effect (η^2^ < 0.01), slight (0.01 ≤ η^2^ < 0.06), moderate (0.06 ≤ η^2^ < 0.14), and strong (η^2^ ≥ 0.14). A negative value means a decrease in surface temperature between the compared time points.

**Table 6:** Thermal response of 21-day-old broiler chickens during acute heat stress *.

Body Region	Mean Temp. Before HS (°C)	SE _Before HS	Mean Temp. During HS (°C)	SE _During HS	Mean Temp. After HS (°C)	SE _After HS	Mean Peak Outcome (°C)	*p*-Value	η^2^	Effect Classification of the Heat Stress
Back	29.10	0.26	27.69	0.41	27.88	0.21	−1.22	0.014	0.51	Strong impact
Breast	27.00	0.13	25.62	0.32	25.36	0.27	−1.64	0.001	0.67	Strong impact
Comb	29.35	0.24	31.15	0.33	31.64	0.56	2.30	0.004	0.60	Strong impact
Drumstick	31.00	0.67	30.11	0.44	30.12	0.43	−0.88	0.414	0.14	Moderate physiological response
Ear	34.92	0.21	34.04	0.26	33.93	0.16	−0.99	0.013	0.51	Strong impact
Eye	33.75	0.11	33.67	0.28	33.90	0.07	0.14	0.673	0.06	Moderate physiological response
Flank	27.32	0.24	25.14	0.30	25.86	0.33	−1.46	0.001	0.71	Strong impact
Head	29.03	0.39	28.10	0.29	28.60	0.16	−0.43	0.124	0.29	Strong impact
Leg	32.42	0.22	33.84	0.39	34.47	0.38	2.05	0.003	0.62	Strong impact
Neck	30.88	0.14	30.42	0.20	30.17	0.26	0.95	>0.001	0.08	Strong impact
Tail	25.56	0.07	23.86	0.38	24.93	0.49	2.71	>0.001	0.15	Strong impact
Thigh	29.89	0.27	27.62	0.15	28.11	0.21	1.29	>0.001	0.05	Strong impact
Wattle	29.84	0.38	31.88	0.52	32.11	0.46	3.73	>0.001	0.27	Strong impact
Wing	35.78	0.42	35.70	0.15	35.97	0.08	0.10	0.814	0.00	Slight effect

* Statistical significance (*p*-value) and effect size (η^2^) are reported based on repeated-measures ANOVA. SE = Standard error. Effect sizes are interpreted using thresholds to classify the physiological impact of heat stress: no effect (η^2^ < 0.01), slight (0.01 ≤ η^2^ < 0.06), moderate (0.06 ≤ η^2^ < 0.14), and strong (η^2^ ≥ 0.14). A negative value indicates a decrease in surface temperature between the compared time points.

**Table 7:** Thermal response of 35-day-old broiler chickens during acute heat stress *.

Body Region	Mean Temp. Before HS (°C)	SE _Before HS	Mean Temp. During HS (°C)	SE _During HS	Mean Temp. After HS (°C)	SE _After HS	Mean Peak Outcome (°C)	*p*-Value	η^2^	Effect Classification of the Heat Stress
Back	24.73	0.14	26.83	0.47	27.81	0.22	3.08	>0.001	0.71	Strong impact
Breast	23.23	0.24	25.22	0.38	25.91	0.18	2.68	>0.001	0.70	Strong impact
Comb	31.71	0.51	35.23	0.24	35.77	0.18	4.06	>0.001	0.80	Strong impact
Drumstick	25.48	0.51	26.05	0.39	27.60	0.28	2.12	0.004	0.41	Strong impact
Ear	32.89	0.14	32.84	0.27	33.50	0.14	0.61	0.044	0.26	Strong impact
Eye	33.16	0.16	34.37	0.12	34.29	0.10	1.13	>0.001	0.73	Strong impact
Flank	24.99	0.23	26.04	0.48	27.24	0.22	2.25	>0.001	0.53	Strong impact
Head	27.72	0.16	28.39	0.19	29.33	0.12	1.61	>0.001	0.71	Strong impact
Leg	34.11	0.34	36.22	0.16	36.97	0.16	2.86	>0.001	0.79	Strong impact
Neck	26.84	0.25	28.73	0.45	29.48	0.19	2.64	>0.001	0.64	Strong impact
Tail	22.82	0.18	23.64	0.44	24.83	0.20	2.01	>0.001	0.52	Strong impact
Thigh	25.15	0.31	26.34	0.47	28.00	0.37	2.85	>0.001	0.57	Strong impact
Wattle	33.20	0.34	35.54	0.25	35.99	0.16	2.80	>0.001	0.76	Strong impact
Wing	33.46	0.42	33.55	0.56	34.20	0.33	0.75	0.453	0.07	Moderate physiological response

* Statistical significance (*p*-value) and effect size (η^2^) are reported based on repeated-measures ANOVA. SE = Standard error. Effect sizes are interpreted using thresholds to classify the physiological impact of heat stress: no effect (η^2^ < 0.01), slight (0.01 ≤ η^2^ < 0.06), moderate (0.06 ≤ η^2^ < 0.14), and strong (η^2^ ≥ 0.14).

**Table 8:** Thermal response of 39-day-old broiler chickens during acute heat stress *.

Body Region	Mean Temp. Before HS (°C)	SE _Before HS	Mean Temp. During HS (°C)	SE _During HS	Mean Temp. After HS (°C)	SE _After HS	Mean Peak Outcome (°C)	*p*-Value	η^2^	Effect Classification of the Heat Stress
Back	24.71	0.17	25.85	0.32	26.16	0.23	1.45	0.001	0.48	Strong impact
Breast	24.22	0.23	24.25	0.20	24.61	0.18	0.39	0.329	0.10	Strong impact
Comb	33.91	0.15	35.48	0.30	35.93	0.19	2.03	>0.001	0.69	Strong impact
Drumstick	24.84	0.37	24.76	0.27	26.09	0.33	1.25	0.013	0.34	Strong impact
Ear	32.80	0.20	33.45	0.24	33.67	0.25	0.87	0.038	0.27	Strong impact
Eye	33.69	0.13	34.66	0.19	34.38	0.23	0.69	0.004	0.41	Strong impact
Flank	24.90	0.17	24.75	0.19	25.98	0.27	1.08	0.001	0.48	Strong impact
Head	27.93	0.09	28.64	0.20	28.89	0.22	0.96	0.003	0.42	Strong impact
Leg	34.49	0.15	36.46	0.17	36.67	0.15	2.17	>0.001	0.85	Strong impact
Neck	26.84	0.25	28.73	0.45	29.48	0.19	1.12	0.020	0.31	Strong impact
Tail	22.82	0.18	23.64	0.44	24.83	0.20	1.55	>0.001	0.54	Strong impact
Thigh	25.15	0.31	26.34	0.47	28.00	0.37	0.66	0.009	0.36	Strong impact
Wattle	33.20	0.34	35.54	0.25	35.99	0.16	2.34	>0.001	0.84	Strong impact
Wing	33.46	0.42	33.55	0.56	34.20	0.33	0.36	0.751	0.03	Moderate physiological response

* Statistical significance (*p*-value) and effect size (η^2^) are reported based on repeated-measures ANOVA. SE = Standard error. Effect sizes are interpreted using thresholds to classify the physiological impact of heat stress: no effect (η^2^ < 0.01), slight (0.01 ≤ η^2^ < 0.06), moderate (0.06 ≤ η^2^ < 0.14), and strong (η^2^ ≥ 0.14).

## Data Availability

Data is available as Supplementary Materials.

## Supplementary Materials

The following supporting information can be downloaded at: https://www.mdpi.com/article/10.3390/ani15152233/s1, Table S1: Thermal response of 14-day-old broiler chickens to acute heat stress; Table S2: Thermal response of 21-day-old broiler chickens during acute heat stress; Table S3: Thermal response of 35-day-old broiler chickens to acute heat stress; Table S4: Thermal response of 39-day-old broiler chickens to acute heat stress.
