# Supplementary material for: Tracking Heat Stress in Broilers: A Thermographic Analysis of Anatomical Sensitivity Across Growth Stages
Source: Animals (Basel). 2025 Jul 29;15(15):2233. doi: 10.3390/ani15152233 (PMC12345529; doi:10.3390/ani15152233)
Supplement: Supplementary file 1 [file animals-15-02233-s001.zip › animals-3750951-supplementary.pdf]

Table S1. Thermal response of 14-day-old broiler chickens to acute heat stress. Mean surface temperatures (°C) of selected anatomical regions before, during, and after heat stress exposure, along with calculated peak temperature variations (minimum, maximum, and mean). Statistical significance (p-value) and effect size (η^2) are reported based on repeated-measures ANOVA. Effect sizes are interpreted using thresholds to classify the physiological impact of heat stress: no effect ($\eta^2 < 0.01$), slight ($0.01 \leq \eta^2 < 0.06$), moderate ($0.06 \leq \eta^2 < 0.14$), and strong ($\eta^2 \geq 0.14$).

Body region	Mean Temp. before HS (°C)	SE _Before	Mean Temp. during HS (°C)	SE _During	Mean Temp. after HS (°C)	SE _After	Min peak increase (°C)	Max peak increase (°C)	Mean peak increase (°C)	p-value	η^2	Effect classification of the heat stress
back	29.708	0.087	30.089	0.111	30.628	0.107	0.538	0.381	0.919	0	0.057	Slight effect
breast	30.366	0.152	29.299	0.231	30.217	0.218	0.918	-1.067	-0.149	0	0.043	Slight effect
comb	30.076	0.256	32.813	0.467	33.073	0.391	0.26	2.738	2.998	0	0.223	Strong impact
drumstick	30.835	0.17	31.139	0.207	32.227	0.164	1.088	0.304	1.393	0	0.075	Moderate physiological response
ear	35.316	0.133	35.4	0.183	35.831	0.13	0.431	0.084	0.516	0.037	0.049	Slight effect of heat stress
eye	34.242	0.121	33.96	0.148	34.836	0.113	0.876	-0.282	0.593	0	0.156	Strong impact
flank	28.564	0.133	26.765	0.121	28.369	0.114	1.605	-1.8	-0.195	0	0.107	Moderate physiological response
head	30.327	0.184	30.35	0.216	30.805	0.149	0.455	0.023	0.478	0.123	0.023	Slight effect
leg	33.889	0.202	35.754	0.163	36.296	0.13	0.541	1.866	2.407	0	0.175	Strong impact
neck	30.853	0.136	30.506	0.15	31.802	0.147	1.297	-0.347	0.949	0	0.075	Moderate physiological response
tail	26.884	0.257	27.79	0.294	29.592	0.315	1.802	0.906	2.708	0	0.145	Strong impact
thigh	31.397	0.17	32.097	0.258	32.688	0.186	0.592	0.7	1.292	0	0.051	Slight effect
wattle	28.107	0.344	30.856	0.504	31.836	0.288	0.98	2.749	3.729	0	0.272	Strong impact
wing	35.242	0.178	35.176	0.18	35.339	0.184	0.163	-0.066	0.097	0.814	0.001	Not meaningful

SE=Standard error. Negative value means a decrease in surface temperature between the compared time points.

Table S2. Thermal response of 21-day-old broiler chickens during acute heat stress. Mean surface temperatures (°C) for anatomical regions recorded before, during, and after exposure to heat stress. Temperature variations (min, max, and mean peaks) are shown alongside p-values and η^2values derived from repeated-measures ANOVA. Effect size categories reflect the physiological intensity of response to heat stress, facilitating a comparison of anatomical thermal sensitivity between developmental stages. Thermal Response to Heat Stress - 21 Days Old Broilers

Body region	Mean_Before HS (°C)	SE_Before	Mean_During HS (°C)	SE_During	Mean_After HS (°C)	SE_After	Min_Peak increase (°C)	Max_Peak increase (°C)	Mean_Peak increase (°C)	p_value	η^2	Effect
back	29.096	0.262	27.693	0.409	27.880	0.208	-0.904	-0.380	-1.216	0.014	0.510	Strong impact
breast	26.998	0.129	25.617	0.322	25.359	0.266	-0.988	-0.413	-1.639	0.001	0.671	Strong impact
comb	29.347	0.244	31.151	0.330	31.642	0.562	-0.622	2.856	2.296	0.004	0.601	Strong impact
drumstick	30.999	0.671	30.107	0.439	30.119	0.430	-2.544	1.511	-0.880	0.414	0.137	Moderate physiological response
ear	34.924	0.214	34.042	0.264	33.933	0.158	-1.100	0.444	-0.991	0.013	0.513	Strong impact
eye	33.753	0.105	33.673	0.282	33.898	0.066	-0.489	0.678	0.144	0.673	0.064	Moderate physiological response
flank	27.322	0.239	25.139	0.300	25.861	0.327	-0.232	-1.106	-1.461	0.001	0.709	Strong impact
head	29.028	0.388	28.097	0.292	28.598	0.159	-0.033	-0.067	-0.430	0.124	0.294	Strong impact
leg	32.417	0.221	33.838	0.385	34.467	0.384	-0.489	3.283	2.050	0.003	0.616	Strong impact
neck	30.881	0.135	30.423	0.200	30.167	0.257	-0.947	0.017	-0.714	0.079	0.345	Strong impact
tail	25.556	0.071	23.858	0.383	24.928	0.491	0.017	-0.861	-0.628	0.019	0.484	Strong impact
thigh	29.887	0.265	27.619	0.153	28.114	0.214	0.246	-1.079	-1.773	0.000	0.836	Strong impact
wattle	29.838	0.379	31.878	0.515	32.113	0.461	-2.478	5.344	2.276	0.008	0.558	Strong impact
wing	35.783	0.415	35.704	0.151	35.970	0.084	-0.290	0.948	0.188	0.761	0.044	Slight effect

SE=standard error. Negative value means a decrease in surface temperature between the compared time points.

Table S3. Thermal response of 35-day-old broiler chickens to acute heat stress. Mean surface temperatures (°C) of each anatomical region before, during, and after heat stress exposure, including standard errors, thermal peak variation metrics, p-values, and effect sizes (η^2). Results were derived using repeated-measures ANOVA. Effect size interpretations classify the intensity of each region's response based on established thresholds.

Body region	Mean_Before HS (°C)	SE_Before	Mean_DuringHS (°C)	SE_During	Mean_After HS (°C)	SE_After	Min_Peak increase (°C)	Max_Peak increase (°C)	Mean_Peak increase (°C)	p_value	η^2	Effect
back	24.729	0.142	26.825	0.469	27.808	0.217	-1.416	4.038	3.079	0.000	0.711	Strong impact
breast	23.233	0.244	25.218	0.379	25.912	0.175	-1.217	3.733	2.679	0.000	0.703	Strong impact
comb	31.714	0.514	35.228	0.242	35.774	0.180	-0.189	5.333	4.060	0.000	0.796	Strong impact
drumstick	25.478	0.510	26.045	0.386	27.600	0.282	-0.607	2.411	2.123	0.004	0.414	Strong impact
ear	32.890	0.144	32.837	0.269	33.504	0.141	-1.356	1.111	0.614	0.044	0.258	Strong impact
eye	33.164	0.159	34.369	0.122	34.293	0.096	-0.878	1.867	1.129	0.000	0.725	Strong impact
flank	24.991	0.228	26.039	0.477	27.238	0.217	-0.724	2.306	2.247	0.000	0.525	Strong impact
head	27.723	0.160	28.386	0.194	29.328	0.119	-0.092	1.400	1.605	0.000	0.706	Strong impact
leg	34.111	0.335	36.224	0.162	36.966	0.160	-0.178	3.231	2.855	0.000	0.792	Strong impact
neck	26.844	0.247	28.733	0.454	29.484	0.187	-2.094	4.061	2.640	0.000	0.636	Strong impact
tail	22.819	0.177	23.635	0.444	24.830	0.201	-0.850	2.528	2.011	0.000	0.521	Strong impact
thigh	25.148	0.305	26.339	0.469	28.000	0.365	-1.156	3.542	2.851	0.000	0.568	Strong impact
wattle	33.196	0.340	35.538	0.252	35.994	0.162	-1.500	3.600	2.799	0.000	0.758	Strong impact
wing	33.457	0.417	33.552	0.564	34.201	0.327	-1.457	2.167	0.745	0.453	0.073	Moderate physiological response

SE=standard error. Negative value means a decrease in surface temperature between the compared time points.

Table S4. Thermal response of 39-day-old broiler chickens to acute heat stress. Temperature data across three phases (before, during, and after stress) are presented for all anatomical regions, with statistical comparisons based on repeated-measures ANOVA. Peak temperature changes and η^2 values highlight the regions most and least affected by heat exposure, offering insight into late-stage thermoregulatory behavior in broilers.

Body region	Mean_Before HS (°C)	SE_Before	Mean_During HS (°C)	SE_During	Mean_After HS (°C)	SE_After	Min_Peak	Max_Peak increase (°C)	Mean_Peak increase (°C)	p_value	η^2	Effect
back	24.711	0.169	25.847	0.318	26.156	0.230	-0.860	2.242	1.445	0.001	0.476	Strong impact
breast	24.217	0.228	24.245	0.198	24.609	0.177	-0.575	1.154	0.392	0.329	0.100	Moderate physiological response
comb	33.907	0.149	35.476	0.296	35.933	0.185	-0.722	2.244	2.026	0.000	0.692	Strong impact
drumstick	24.837	0.366	24.757	0.265	26.092	0.330	-0.252	2.389	1.254	0.013	0.338	Strong impact
ear	32.801	0.198	33.450	0.243	33.671	0.248	-0.233	1.444	0.869	0.038	0.267	Strong impact
eye	33.694	0.125	34.658	0.190	34.382	0.226	-1.733	1.722	0.688	0.004	0.406	Strong impact
flank	24.895	0.174	24.753	0.186	25.980	0.273	-0.501	1.225	1.084	0.001	0.479	Strong impact
head	27.933	0.093	28.635	0.200	28.892	0.218	-0.967	1.442	0.958	0.003	0.423	Strong impact
leg	34.494	0.154	36.456	0.167	36.665	0.153	-0.872	2.775	2.170	0.000	0.845	Strong impact
neck	26.802	0.206	27.705	0.320	27.923	0.278	-1.231	1.908	1.121	0.020	0.313	Strong impact
tail	22.889	0.133	23.085	0.255	24.439	0.305	-0.717	1.133	1.550	0.000	0.536	Strong impact
thigh	25.440	0.234	24.973	0.241	26.104	0.227	0.275	0.658	0.664	0.009	0.360	Strong impact
wattle	33.878	0.188	35.900	0.171	36.221	0.154	-0.178	3.244	2.343	0.000	0.839	Strong impact
wing	31.354	0.245	31.672	0.438	31.717	0.392	-2.752	1.829	0.364	0.751	0.027	Slight effect

SE=standard error. Negative value means a decrease in surface temperature between the compared time points.
